# Assessment of Variation in US State Laws Addressing the Prevention of and Response to Teen Dating Violence in Secondary Schools

**DOI:** 10.1001/jamapediatrics.2022.1735

**Published:** 2022-06-13

**Authors:** Avanti Adhia, Melanie Kray, Deirdre Bowen, Mary A. Kernic, Elizabeth Miller

**Affiliations:** 1 Department of Pediatrics, School of Medicine, University of Washington, Seattle; 2 Harborview Injury Prevention and Research Center, School of Medicine, University of Washington, Seattle; 3 University of Washington School of Law, Seattle; 4 Seattle University School of Law, Seattle, Washington; 5 Department of Epidemiology, School of Public Health, University of Washington, Seattle; 6 Division of Adolescent and Young Adult Medicine, University of Pittsburgh Medical Center Children’s Hospital of Pittsburgh, Pittsburgh, Pennsylvania

## Abstract

**Importance:**

Teen dating violence (TDV) is widespread and has adverse consequences across the life course. Many US states are actively seeking strategies to prevent and address TDV in schools, including enactment and implementation of TDV laws. These state laws are upstream, system-level interventions that lay the foundation for an array of downstream targeted interventions to be deployed in schools. Despite these laws gaining momentum in the past decade, there has been minimal research examining their content and variability.

**Objective:**

To assess the content and comprehensiveness of US state laws addressing prevention of and response to TDV in secondary schools.

**Design, Setting, and Participants:**

A team of public health and legal researchers used systematic policy surveillance methods to develop a comprehensive database of state laws addressing TDV in schools in all 50 US states and the District of Columbia (referred to hereinafter as “states”) in effect as of September 30, 2020. Using LexisNexis and state legislature websites, state laws (statutes and administrative codes) were identified.

**Main Outcomes and Measures:**

Components for each law were coded within 5 categories: scope, prevention education, policy, response, and implementation.

**Results:**

Overall, 38 states (74.5%) had at least 1 law addressing TDV in secondary schools and 13 states (25.5%) did not. Fewer than half of states with a law (15 of 38 [39.5%]) defined TDV. All 38 states addressed prevention education, with 29 (76.3%) requiring and 9 (23.7%) encouraging these efforts. Prevention education was largely for students in most states (35 [92.1%]), but some states also required school staff (18 [47.4%]) and parents (8 [21.1%]) to receive prevention education. Fewer than half of states required (14 [36.8%]) or encouraged (3 [7.9%]) school districts to develop a policy addressing TDV. Very few states included requirements for how schools must respond to TDV incidents. Fewer than a third of states designated an individual to coordinate TDV efforts (12 [31.6%]), included a funding provision in their laws for TDV programs and policies (10 [26.3%]), and included explicit consequences for noncompliance with TDV laws (12 [31.6%]).

**Conclusions and Relevance:**

This cross-sectional study found that most US states had laws addressing TDV in schools, but there was significant variation in the content and comprehensiveness of the laws. These findings can inform the development of more comprehensive laws on TDV that may ultimately reduce such incidents.

## Key Points

**Question:**

How variable are US state laws addressing the prevention of and response to teen dating violence in secondary schools?

**Findings:**

In this cross-sectional surveillance study of 50 US states and the District of Columbia, 38 states (74.5%) had at least 1 law addressing teen dating violence in secondary schools as of September 30, 2020, and 13 states (25.5%) did not. There was considerable variation in components related to prevention education and development of school district policies; for example, fewer than a third of states included a funding provision or consequences for noncompliance.

**Meaning:**

The findings of this study suggest that although most states have laws addressing teen dating violence in secondary schools, they could benefit from additional provisions to more clearly guide schools, including resources and incentives to implement these laws.

## Teaser

This cross-sectional study assesses the content and comprehensiveness of state laws that address the prevention of and response to teen dating violence among students in US secondary schools.

## Introduction

Despite an increased focus on prevention, intimate partner violence remains a substantial public health problem. Adolescents and young adults are disproportionately affected, as reflected in public health survey data.^1^ Among US high school students who dated, 12.2% experienced physical violence, sexual violence, or both by someone they were dating or going out with in the past year.^2^ In a nationally representative survey of 12- to 18-year-old youths who dated, more than 60% experienced psychological or emotional abuse.^3^ Furthermore, the prevalence of technology-assisted or cyber abuse among adolescents in dating relationships has been estimated to be as high as 75.0%.^4^ These experiences of teen dating violence (TDV) can have health consequences across the life course, including depression, suicidal ideation, increased substance use, experiencing intimate partner violence as an adult, and, in extreme cases, death.^5,6^

Creating protective community environments has been identified as a key strategy for preventing TDV.^7^ Schools are an ideal setting for TDV prevention because they contain a wide and diverse audience, offer consistent interaction with adolescents, are where youths spend most of their time outside the home, and are where many aspects of adolescent relationships take place.^8^ School environments that foster connectedness and TDV awareness can create a climate that encourages care seeking, increases support for affected students, and promotes social norms intolerant of TDV.^7,8^ Changing or adapting school environments as part of a multilevel strategy, including educational programs and physical environment modifications, has been reported to reduce TDV.^9^

To date, with rare exceptions,^9^ TDV interventions have largely focused on individual and relationship-level factors and can be challenging to implement widely.^10,11^ However, there have been calls to prioritize community and societal strategies addressing the outer layers of the social ecology to increase population-level impact.^12^ Laws addressing TDV in schools are among the latest developments in broad, system-level interventions that US states are enacting to address interpersonal violence within school settings.^13^ These laws are upstream interventions that lay the foundation for an array of downstream targeted interventions to be deployed in schools (eg, training for school personnel, integration of educational curricula, or provision of counseling).^14,15^ Previous studies report that interventions focused on the whole school are more effective than individual-level interventions delivered through classroom curricula or social skills training alone.^9,16^

Similar to antibullying legislation, TDV laws have the potential to alter the social context in which TDV occurs by encouraging or requiring school districts to adopt prevention education curricula and policies for addressing TDV.^13,17^ Currently, there is no national model or language that is applied consistently across states; for example, some states require schools to simply have a written policy for responding to TDV, whereas others additionally require implementation of TDV prevention programming.^13^ Despite these laws gaining momentum in the past decade, to date there has been minimal research examining their content and variability.

This study aimed to assess the content and variability of US state laws addressing the prevention of and response to TDV in secondary schools. Policy surveillance (the systematic collection, tracking, and analysis of laws over time) is foundational to the rigorous evaluation of laws and policies that promote public health.^18^ Although this study focused on the most recent status of state TDV laws, the results are part of a larger effort to create a longitudinal, empirical database of these laws that tracks variation over time. This effort aims to (1) enable investigation of the association of these laws with TDV prevalence and (2) provide an ongoing resource that can be updated to track future changes in these laws.

## Methods

This cross-sectional study was deemed exempt from review by the University of Washington Institutional Review Board because it was not considered human participants research. The study followed the Strengthening the Reporting of Observational Studies in Epidemiology (STROBE) reporting guideline.

Using legal databases and state legislature websites, an interdisciplinary team of public health and legal researchers conducted a systematic assessment of laws related to TDV in schools across all 50 US states and the District of Columbia (referred to hereinafter as “states”). Laws included statutes and administrative codes/regulations in states and reflect information in effect as of September 30, 2020. Two prior sources from the National Association of State Boards of Education^13^ and the National Conference of State Legislatures^19^ served as a starting point. We developed a detailed protocol with search terms, a codebook, and coding rules that documented how we applied codes to maintain consistency.^20^

To locate relevant laws, a legal researcher (M.K.) used the following keywords as search terms in LexisNexis: *dating violence and schools*, *dating abuse and schools*, *abusive relationships*, *sexual violence in dating*, *domestic violence education*, *teen dating violence*, *health education*, and *healthy relationships*. Keyword searches were supplemented by examining the table of contents of each relevant section and performing additional Westlaw database searches as needed. We included laws explicitly related to secondary schools and adolescents that had 1 of the following TDV-related terms: *dating violence or abuse*, *relationship violence or abuse*, *partner violence or abuse*, *domestic violence or abuse*, *abusive relationships*, or *healthy relationships*. Laws that focused exclusively on colleges and universities or illicit conduct handled by juvenile or criminal courts, that were more localized than administrative codes, or that did not explicitly mention schools and adolescents were excluded. Laws related to sexual harassment, sexual violence, or bullying were included only if they also explicitly referenced violence among adolescents within dating or intimate relationships.

We created an initial codebook based on the following: (1) the framework established for content of antibullying laws^21,22^; (2) a model school policy created by Futures Without Violence, a national nonprofit focused on interpersonal violence^23^; (3) laws from a random sample of 5 states; and (4) the legal and substantive knowledge of the research team and consultants. We entered the coding questions into MonQcle, a web-based software platform designed for researchers to create legal data sets.^24^ We sought to avoid subjectivity by relying on the plain text of the laws rather than interpreting their meaning. To keep coding as objective as possible, a component was coded “no” if the information was not explicitly specified in the law. Two researchers (A.A. and M.K.) followed an iterative process of refining the coding questions and procedures.^20^

We coded the following categories and components of TDV laws: scope of TDV law, TDV prevention education, TDV policy, response to TDV, and implementation of TDV policy or prevention. A detailed description of all variables is provided in the eMethods in the Supplement.

To ensure data quality, 2 researchers (A.A. and M.K.) independently coded the collected laws for a subset of states.^20^ To refine the coding scheme, the coding process was completed in batches of 5 states. We redundantly coded all states in the first 2 batches, and then 1 state per set of 5 after the first 2. In total, 18 states with a TDV law (47.4%) were redundantly coded, and the mean divergence rate between the 2 researchers across all coding questions was 10.3%. Team members documented issues that arose during the coding process and iteratively revised and reapplied the coding scheme, consulting with the full team as needed. For each component, we generated the number and percentage of states that included the component in their TDV laws.

## Results

Overall, 38 states (74.5%) had at least 1 law explicitly addressing TDV prevention and response in secondary schools, and 13 (25.5%) did not (Figure). Among the states with laws addressing TDV, there was considerable variability across the components (Table). eTables 1 and 2 in the Supplement provide data for each state.

**Figure.**
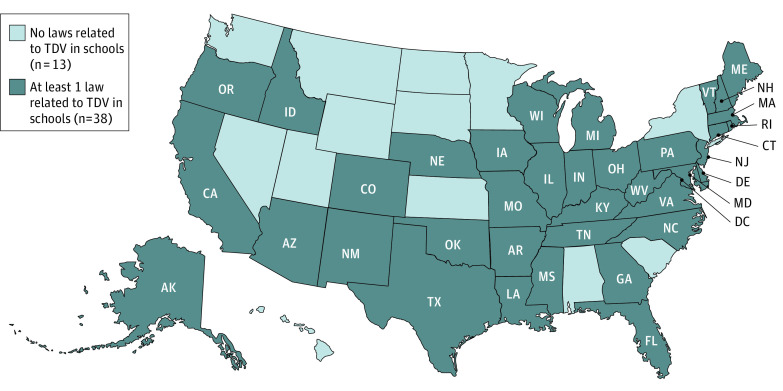
Laws Addressing Teen Dating Violence in Secondary Schools in the 50 US States and the District of Columbia as of September 30, 2020 TDV indicates teen dating violence.

**Table.** Components of Laws Addressing Teen Dating Violence in Secondary Schools in 38 US States^a^ as of September 30, 2020

Component of state law	No. of US states with TDV laws (%)
**Scope of TDV law**	
Defines TDV	
No	23 (60.5)
Yes	15 (39.5)
Addresses cyber TDV	
No	29 (76.3)
Yes	9 (23.7)
Grade levels included^b^	
Not specified	5 (13.2)
High school only	3 (7.9)
High school plus middle school only	13 (34.2)
Kindergarten to 12th grade	17 (44.7)
**TDV prevention education**	
Prevention education included	
No	0 (0.0)
Yes, encouraged	9 (23.7)
Yes, required	29 (76.3)
Content of prevention education^b^	
Not specified	3 (7.9)
Healthy relationships	31 (81.6)
Awareness education	22 (57.9)
Definition of TDV	15 (39.5)
Warning signs	14 (36.8)
Bystander education	10 (26.3)
Prevention education based on external standards	
No	15 (39.5)
Yes, external standard	9 (23.7)
Yes, evidence based	14 (36.8)
Prevention education subject to external review	
No	21 (55.3)
Yes (eg, by state, district, or external organization)	17 (44.7)
Who must be trained on prevention education^b^	
Students	35 (92.1)
School staff (eg, administration, teachers)	18 (47.4)
Parents	8 (21.1)
**TDV policy**	
School districts must develop TDV policy	
No	21 (55.3)
Yes, encouraged	3 (7.9)
Yes, required	14 (36.8)
Where policy applies^b^^,^^c^	17
School property	17 (100.0)
School transportation	7 (41.2)
School event	7 (41.2)
Electronic communication	3 (17.6)
Policy subject to external review^c^	17
No	9 (52.9)
Yes (eg, by state, district, or external organization)	8 (47.1)
Who must be trained on the policy^b^^,^^c^	17
No training	5 (29.4)
Students	8 (47.1)
School staff (including teachers or administration)	11 (64.7)
Parents	8 (47.1)
**Response to TDV**	
Investigation requirements once TDV is reported	
No	34 (89.5)
No, but districts must develop in policy	2 (5.3)
Yes	2 (5.3)
Disciplinary consequences for perpetrating TDV	
No	28 (73.7)
No, but districts must develop in policy	10 (26.3)
Yes	0 (0.0)
Mental health services for affected students	
No	35 (92.1)
Yes	3 (7.9)
**Implementation of TDV policy or prevention**	
Designated individual for TDV efforts	
No	26 (68.4)
Yes	12 (31.6)
Funding provision	
No	28 (73.7)
Yes (eg, for prevention programs, policy training)	10 (26.3)
Consequences for noncompliance	
No	26 (68.4)
Yes	12 (31.6)

Abbreviation: TDV, teen dating violence.

^a^ Includes the District of Columbia.

^b^ Because states can be included in multiple categories, percentages sum to more than 100%.

^c^ Percentage calculated only out of those 17 states with laws that school districts must have a TDV policy.

### Scope of TDV Law

According to our results, 15 states (39.5%) defined TDV, and 9 states (23.7%) addressed cyber TDV. In 5 states (13.2%), the grade levels covered by the law were not specified. Three states (7.9%) included high school only, 13 (34.2%) included high school and middle school only, and 17 (44.7%) included kindergarten through 12th grade.

### TDV Prevention Education

All 38 states addressed prevention education, with most requiring (29 [76.3%]) and some encouraging (9 [23.7%]) such efforts. Prevention education most commonly included healthy relationships (31 [81.6%]) and awareness education (22 [57.9%]). Fewer than half of states covered the definition of TDV in their prevention education (15 [39.5%]) or taught about TDV warning signs (14 [36.8%]). Only about a quarter of states included bystander education (10 [26.3%]).

Approximately a third of states (14 [36.8%]) required evidence-based prevention education, whereas another 9 (23.7%) required it to be based on some other external standard (eg, consultation with a community organization). Fewer than half of states (17 [44.7%]) required prevention education to be subject to external review. Nearly all states (35 [92.1%]) required students to receive prevention education, whereas 18 states (47.4%) required it for school staff, and 8 states (21.1%) required it for parents.

### TDV Policy

Fewer than half of states required (14 [36.8%]) or encouraged (3 [7.9%]) school districts to develop a policy addressing TDV. In all 17 states (100%) where policies were encouraged or required, the policy consistently applied if TDV occurred on school property. Seven states (41.2%) additionally stated that the policy would apply to school transportation, 7 (41.2%) included school events, and 3 (17.6%) included electronic communication. Eight states (47.1%) that included TDV policies in their laws mandated that they be subject to external review either by a state entity, their local school district, or some other external organization. Although nearly a third of states (5 [29.4%]) did not require any policy training, 11 states (64.7%) required school staff to be trained or informed about the policy, 8 states (47.1%) required it for parents, and 8 states (47.1%) required it for students.

### Response to TDV

Overall, very few states included any requirements for how schools must respond to TDV incidents. Only 2 states (5.3%) mandated specific investigation requirements once TDV was reported, and 2 states (5.3%) mandated that school districts develop investigation requirements in their TDV policy. No states included specific disciplinary consequences for those perpetrating TDV, and 10 states (26.3%) mandated that school districts develop disciplinary consequences in their TDV policy. Only 3 states (7.9%) provided mental health services for students affected by TDV.

### Implementation of TDV Policy or Prevention

Fewer than a third of states (12 [31.6%]) designated an individual or required or encouraged schools or districts to designate one to coordinate TDV efforts. Approximately one-quarter of states (10 [26.3%]) included a funding provision in their laws for TDV programs and policies. Only 12 states (31.6%) included explicit consequences for noncompliance with TDV-related laws.

## Discussion

Across the US, state legislatures have been responding to increasing public awareness and recognition of the adverse consequences of TDV by enacting legislation to address it in schools. These laws have the potential to prevent abusive behaviors and to reduce adverse consequences among youths involved in TDV.^22^ Notably, 13 states (25.5%) in this cross-sectional study had not enacted any laws addressing TDV in schools. Research on the enactment of TDV laws could shed light on modifiable political and social factors to promote passage of more effective TDV legislation. Such research may help identify why and how states choose to enact these laws. Among states with laws addressing TDV in schools, our results highlight the significant variation and gaps in the content and comprehensiveness of these laws. Therefore, additional research is needed to sharpen the focus on effective TDV laws, which in turn may increase the likelihood of enactment.

Regarding the scope of TDV laws, the majority of states in this study did not define TDV or address cyber or technology-assisted TDV. A clear definition and enumeration of specific behaviors sets the foundation for subsequent laws, especially given the overlap in definitions, behaviors, and outcomes of TDV, bullying, and sexual harassment.^17^ The lack of a clear definition can make it challenging for school staff to determine when to respond and to know when other policies may be involved (eg, Title IX). In setting the scope, laws did not explicitly acknowledge the increased risk for experiencing TDV among youths from marginalized groups.^25^ Just as antibullying laws often enumerate protected groups, TDV laws could benefit from recognizing that prevention and response may require culturally specific strategies.^21,26^ As noted earlier, most states’ laws did not mention cyber or technology-assisted abuse; this raises important concerns about whether and how schools should respond to cyber TDV, given the prevalence and importance of technology for adolescents.^4^ Also relevant to scope are the grade levels included. Although many states included relevant education in kindergarten through 12th grade, some states included only middle schools (grades 5-8) or high schools (grades 9-12) in TDV-specific laws. Most TDV prevention programs target high school–aged youths, despite recognition that early adolescence is an important window for intervention.^9,25^ Schools can introduce foundational socioemotional learning and boundary-setting concepts in age-appropriate ways for younger students, while building to concepts of violence and abuse in adolescent years.^7,25^

In this study, all states with TDV laws included some form of prevention education, largely for students. The content of required education was vague in most laws, and many states did not require the education to be based on any standards or subject to external review, allowing for wide variance in interpretation and implementation. More guidance regarding evidence-based approaches (eg, skill-building, including practice discussing disagreements appropriately) could ensure that a baseline standard is met in statewide public education while allowing schools to tailor programs to their students’ needs.^8,27^ Bystander education was the least common component of education specified in the laws, despite promising evidence that it can increase youths’ willingness to intervene if they witness TDV.^28^ By focusing on collective responsibility, bystander education can also establish social norms around intolerance for violence, contributing to a more supportive school climate.^28,29^ Importantly, state laws did not necessarily specify who should be providing prevention education, which could influence the effectiveness of such programs.

Fewer than half of states with TDV laws encouraged or required school districts to develop a written TDV policy. Research on antibullying laws suggests that laws requiring districts to develop and implement local policies are effective in reducing bullying.^22^ In addition to requiring TDV policies, policy content must be carefully considered. In some states, the department of education develops a model TDV policy for districts to adopt or adapt. Futures Without Violence created a model policy to increase safety and improve school climate.^23^ Although TDV has specific characteristics, antibullying policies can serve as a useful starting point; in addition, school districts could address bullying and TDV in an integrated policy, given the overlap between these behaviors.^17,30^ Among states that included TDV policies in their laws, almost a third did not require anyone to be trained on or informed of the policy. For written policies to be implemented, dissemination to staff, students, and parents is critical and can be achieved both through educational sessions and through inclusion in standards of conduct, school handbooks, and school rules.^22,31^ Additional research on promising communication practices could help ensure that staff, students, and parents receive and understand TDV policies.^32,33^

Without clear and comprehensive policies and procedures, schools may not effectively respond to incidents of TDV. In this study, very few state laws included guidance about investigating incidents once TDV has been reported. Such guidance should encourage prompt responses and actions that protect students from experiencing additional violence or retaliation. In addition, approximately one-quarter of states with TDV laws mandated that districts develop disciplinary consequences in their TDV policy. Antibullying research suggests that school-mandated disciplinary actions can deter and reduce bullying.^34^ Response to TDV should address the counseling and mental health needs of students who experience or use TDV. Although some states in this study required schools to provide students information or referrals to community organizations, very few required schools or districts to provide services themselves. Students may not disclose TDV to their caregivers or may lack access to their own transportation and resources, so the ability to access such services at school is important in addressing the psychological effects of TDV.^5,35^

School districts require resources and incentives to implement education, policy, and response components in the face of competing demands and priorities. The general lack of individuals designated by law to oversee implementation of TDV efforts—compounded in most cases by a lack of funding—that we observed in this study raises concerns that laws mandating curriculum or policy alone are insufficient to meaningfully affect TDV behaviors or prevalence.^8,32,33^ Without funding or a designated individual to oversee TDV efforts, implementation may inadvertently go unaddressed. TDV laws also generally lack sanctions or incentives, leaving implementation and enforcement in question. In this study, only 12 states (31.6%) specified possible consequences (eg, withholding state funding from the school district) for noncompliance with the law. The lack of accountability for implementation and enforcement in states’ laws points to a need for research examining whether and how these laws are being carried out in practice.^32,33^

### Limitations

Our findings must be considered in light of several limitations. This was a point-in-time assessment of state laws that were in effect as of September 30, 2020 and are amended frequently. We only examined the text of the laws and had no information on how they were interpreted by schools and districts or what was being implemented at that time. Importantly, the implementation of state laws and school policies is heavily influenced by a number of factors such as community norms, school board leadership, and access to resources, which should be examined in future research. Additional studies are also needed to understand how state laws affect TDV prevalence and, specifically, which components of these laws are associated with reduced TDV. This empirical evidence could also bolster efforts to secure additional resources and funding to support implementation and enforcement of laws addressing TDV.

## Conclusions

State laws addressing TDV in secondary schools reflect a growing consensus about the critical role of schools in addressing TDV. Many states have enacted some laws related to TDV, but substantial variability exists in what states deem necessary to include in these laws, leading to gaps in comprehensiveness. The gaps identified in this cross-sectional study underscore the need for advocacy efforts—including those by pediatricians, who may regularly see adolescent patients experiencing TDV—to enact, improve, and implement these laws. For example, the American Academy of Pediatrics and other major health organizations may be able to guide the development of a model policy or provide recommendations to policy makers regarding key components to include in state laws addressing TDV. Although multiple services are needed to prevent and respond to TDV outside of school, future research should investigate whether these state laws addressing TDV in schools can be an important part of reducing its prevalence. Our findings can support advocates and policy makers in considering specific components of TDV laws to include and can inform the development of more comprehensive laws that may ultimately lead to reduced TDV.

## Notes

Supplement. **eMethods.** Detailed Coding Protocol for US State Laws Addressing Teen Dating Violence in Schools as of September 30, 2020 **eTable 1.** Scope of State Laws and Prevention Education Addressing Teen Dating Violence in Secondary Schools in 50 US States and the District of Columbia as of September 30, 2020 **eTable 2.** State Laws Addressing Teen Dating Violence Policy and Response and the Implementation of Teen Dating Violence Policy or Prevention in Secondary Schools in 50 US States and the District of Columbia as of September 30, 2020
