# Supplementary material for: Assessment of Variation in US State Laws Addressing the Prevention of and Response to Teen Dating Violence in Secondary Schools
Source: JAMA Pediatr. 2022 Jun 13;176(8):797–803. doi: 10.1001/jamapediatrics.2022.1735 (PMC9194746; doi:10.1001/jamapediatrics.2022.1735)
Supplement: Supplement. — eMethods. Detailed Coding Protocol for US State Laws Addressing Teen Dating Violence in Schools as of September 30, 2020 eTable 1. Scope of State Laws and Prevention Education Addressing Teen Dating Violence in Secondary Schools in 50 US States and the District of Columbia as of September 30, 2020 eTable 2. State Laws Addressing Teen Dating Violence Policy and Response and the Implementation of Teen Dating Violence Policy or Prevention in Secondary Schools in 50 US States and the District of Columbia as of September 30, 2020 [file jamapediatr-e221735-s001.pdf]

Supplementary Online Content

Adhia A, Kray M, Bowen D, Kernic MA, Miller E. Assessment of variation in US state laws addressing the prevention of and response to teen dating violence in secondary schools. *JAMA Pediatr*. Published online June 13, 2022. doi:10.1001/jamapediatrics.2022.1735

eMethods. Detailed Coding Protocol for US State Laws Addressing Teen Dating Violence in Schools as of September 30, 2020
eTable 1. Scope of State Laws and Prevention Education Addressing Teen Dating Violence in Secondary Schools in 50 US States and the District of Columbia as of September 30, 2020
eTable 2. State Laws Addressing Teen Dating Violence Policy and Response and the Implementation of Teen Dating Violence Policy or Prevention in Secondary Schools in 50 US States and the District of Columbia as of September 30, 2020

This supplementary material has been provided by the authors to give readers additional information about their work.

© 2022 Adhia A et al. *JAMA Pediatrics.*

eMethods. Detailed Coding Protocol for US State Laws Addressing Teen Dating Violence in Schools as of September 30, 2020

We coded the following categories and components of TDV laws:

1. *Scope of TDV law*: whether the law included a definition of TDV, whether the law addressed cyber or technology-assisted TDV, and grade levels included;
2. *TDV prevention education*: whether prevention education was encouraged ("may") or required, the content included (eg, healthy relationships, awareness education, TDV definition, warning signs, bystander education), whether prevention education was based on external standards (eg, evidence based or other accepted standard/best practice), whether prevention education was subject to external review (eg, by state entity, local school district, or external organization), and who received training on prevention education (eg, students, staff, parents);
3. *TDV policy*: whether development of a TDV policy by school districts was encouraged or required, where the policy applied (eg, school property, school transportation, school event, electronic communication), whether the policy was subject to external review, and who received training or information about the policy;
4. *Response to TDV*: whether the law specified investigation requirements once TDV was reported, whether the law included disciplinary consequences for perpetrating TDV, and whether mental health services were provided for affected students by the school, district, or state; and
5. *Implementation of TDV policy or prevention*: whether the law designated an individual (or requires or encourages schools and/or districts to designate an individual) to coordinate TDV efforts, whether there was a funding provision (eg, for TDV-related prevention programs, policy training, or policy implementation), and whether there were explicit consequences for noncompliance (eg, withholding or reducing funding, citation, required for accreditation).

General Coding Notes:

- "Family violence" is excluded from coding unless it is linked to broader relationship skills/healthy relationships/abusive relationships
- "Domestic Violence" is excluded from coding unless it is explicitly linked to teenagers/adolescents or schools
- "Positive relationships" is excluded from coding unless it is explicitly linked to teen dating violence (TDV)
- If an administrative regulation only incorporates learning standards (or learning objectives, academic standards, etc.) by reference but does not add anything substantive to the standards, it is not included in sources – only the incorporated learning standards are included

Variable	Coding Question	Response Options	Coding Notes
**Scope of TDV Law**			
Defines TDV	Does the law define TDV?	No<br>Yes	
Addresses cyber TDV	Does the law address cyber or technology-assisted TDV?	No<br>Yes	Yes if law included regulation of/policies addressing, e.g., "sharing of sexually explicit images without permission" or "revenge porn"; the dangers of sexual exploitation on the internet (even if statute did not explicitly say done by dating/intimate partner); or the impact of technology on sexuality/relationships/personal safety/communication skills<br><br>No if law only included regulation of/policies for cyber-bullying or if "cyber" part of statute was not explicitly tied to TDV (mandating that TDV be "included in and consistent with" larger bullying policy counts as explicit tie)

© 2022 Adhia A et al. *JAMA Pediatrics.*

Grade levels included	Which grade levels are included in the law?<br>*Check all that apply.*	K-12<br>Elementary school<br>Middle school<br>High school<br>Grade level not specified	For summary, created mutually exclusive categories: Not specified, high school only, high school + middle school only, and K-12<br><br>Coded based on grades listed in specific statute addressing TDV – i.e., if one statute regulates TDV prevention program and another statute incorporates that prevention program into a broader health education statute, only coding for grades listed in former statute<br><br>Middle school: includes grades above 5th<br><br>High school: includes grades above 9th
**TDV Prevention Education**			
Prevention education included	Does the law include prevention programs?	No<br>Yes, encouraged<br>Yes, required	Yes, required: if, in relation to training on school's TDV policy or educational program, law says "shall," "will," or "must", and mentions "prevention," "warning signs," "red flags," "healthy boundaries," or "healthy relationships"<br><br>Yes, encouraged: if, in relation to training on school's TDV policy or educational program, statute says "may," "encouraged," or "should," and mentions "prevention," "warning signs," "red flags," "healthy boundaries," or "healthy relationships"<br><br>If one statute uses "encouraged" language but another uses "required" language, coded as required
Content of prevention education	What is included in prevention programs?<br>*Check all that apply.*	Not specified<br>Healthy relationships<br>Awareness education<br>Definition<br>Warning signs<br>Bystander education	Healthy relationships: includes "relationship skills," "communication skills," "conflict resolution skills," or generally skills training that contributes to healthy, non-abusive relationships<br><br>Awareness education: mentions "raising awareness," "informing," "identifying," or "recognizing" TDV<br><br>Definition: includes laws that mention teaching about "definition," or "characteristics of [abusive relationships/TDV/healthy relationships]"<br><br>Warning signs: includes "early intervention" as well as explicit mention of "warning signs"<br><br>Bystander education: mentions "bystander," "interven[-ing or -tion]", "recognizing responsibility to report behavior," community or social responsibility, persuading or supporting others to engage in behaviors that reduce violence, showing disapproval of others' behaviors, or helping someone in an abusive relationship

© 2022 Adhia A et al. *JAMA Pediatrics.*

Prevention education based on external standards	Is the prevention program based on external standards?	No<br>Yes, external standard<br>Yes, evidence-based	External standard: includes some acknowledgment of external standards that the program should incorporate or adhere to, such as best practices according to a domestic violence organization, “nationally accepted standards,” or some governmental or non-profit entity schools or districts must consult when creating their program. This does not include local parent-teacher organizations or “education stakeholders”<br><br>Evidence-based: included the terms “evidence based,” “research-based,” or “medically accurate”
Prevention education subject to external review	Must the prevention program be subject to external review?	No<br>Yes (by state, district, external organization)	No unless statute mentioned “submitting for assessment” or being reviewed
Who must receive prevention education	Who must receive prevention programs? *Check all that apply.*	Students<br>Administrators<br>Staff<br>Teachers<br>Parents	For students: included if taught as part of an educational program<br><br>For parents: included if parents were mentioned in reference to prevention (e.g., information had to be provided to parents, “parent awareness training” or “parent notices” specific to TDV)<br><br>School staff included administrators, staff (e.g., nurses, psychologists, other staff), and/or teachers
**TDV Policy**			
School districts must develop TDV policy	Does the law include the development of an TDV policy by school districts?	No<br>Yes, encouraged<br>Yes, required	Yes, required: if law says “shall,” “will,” or “must”, and mentions a policy or rule for addressing incidents of TDV<br><br>Yes, encouraged: if law says “may,” “encouraged,” or “should,” and mentions a policy or rule for addressing incidents of TDV<br><br>If one statute uses “encouraged” language but another uses “required” language, coded as required
Where policy applies	Where does the policy apply? *Check all that apply.*	School property<br>School transportation<br>School event<br>Electronic communication	Coded school property unless otherwise specified
Policy subject to external review	Must the district policy be subject to external review?	Yes (by state, district, external organization)	No unless statute mentioned “submitting for assessment” or being reviewed; only counted review after creation of policy – does not include consultation before or while creating the policy
Who must be trained on or be	Who must be trained on or informed about the	No training<br>Students	For summary, school staff included administrators, staff (e.g., nurses, psychologists, other staff), and/or teachers

© 2022 Adhia A et al. *JAMA Pediatrics.*

informed of the policy	policy? *Check all that apply.*	Administrators<br>Staff<br>Teachers<br>Parents	Counts as training if, in relation to training on school's TDV policy (not educational program, unless training on school's TDV policy is explicitly part of educational program), statute says "shall," "will," "must," "may," "encouraged," or "should." If notice of policy is in district materials/publications that set forth rules and standards of conduct (e.g., school handbook), it is counted as training for whoever the notice is distributed to<br><br>No training if policy was just made available upon request (e.g., to parents) but no additional notification or training was offered. No if statute mentioned more general wellness or health policy with no link to TDV
**Response to TDV**			
Investigation requirements once TDV is reported	Does the law include investigation requirements once TDV is reported?	No<br>No, but school district must develop investigation requirements in policy<br>Yes	No, but school districts must develop: if law mandated investigation of TDV reports but did not specify what investigations must include<br><br>Yes: if law specified what investigations must include
Disciplinary consequences for perpetrating TDV	Does the law include disciplinary consequences?	No<br>No, but school district must develop disciplinary consequences in policy<br>Yes	No, but school districts must develop: if law mandated there be some disciplinary consequences for confirmed TDV incidents but did not specify what consequences must include/be<br><br>Yes: if law included specific disciplinary consequences for confirmed TDV incidents
Mental health services for affected students	Are mental health services provided for victims and/or perpetrators of TDV?	No<br>Yes	Yes: if law included mental health services for "affected students" or for victims offered by the school, district, or state<br><br>No: if statute only included informing students about therapeutic victims services orgs/resources that are not offered by the school, district, or state or if it is unclear who is providing the counseling
**Implementation of TDV Policy or Prevention**			
Designated individual for TDV efforts	Does the law have schools/districts designate an individual to coordinate TDV efforts?	No<br>Yes	Yes: if law says school/district may or must designate someone or create a position (whether or not the term "specialist" is used) to oversee implementing the policy or the educational program OR if statute/act explicitly designates a certain staff position or government official to oversee implementing the policy<br><br>No: if state advisory council or board was to make recommendations about programs or policies, but they were not involved in implementation in schools/districts
Funding provision	Does the law include a funding provision? *Check all that apply.*	No	For summary, combined all funding types

© 2022 Adhia A et al. *JAMA Pediatrics.*

		Yes (for TDV policy, policy training, and/or for prevention programs)	Yes: if explicitly stated or statute has requirements for programs/policies “funded by the State”
Consequences for non-compliance	Are there explicit consequences for non-compliance?	No<br>Yes	Yes: if explicit penalties for non-compliance (e.g., withholding/reduced funding, citation, required for accreditation)<br><br>No: if no explicit penalties for non-compliance – if implicit, not sufficient to code “yes” (e.g., some external body will review the policy or educational program but statute does not identify any consequence if standards aren’t met)

© 2022 Adhia A et al. *JAMA Pediatrics.*

eTable 1. Scope of State Laws and Prevention Education Addressing Teen Dating Violence in Secondary Schools in 50 US States and the District Of Columbia as of September 30, 2020

	Scope of TDV Law			TDV Prevention Education				
**State**	**Defines TDV**	**Addresses cyber TDV**	**Grade levels[a]**	**Prevention education**	**Prevention content[b]**	**External standards**	**External review**	**Who must receive prevention education**
**Alabama**								
**Alaska**	✓	-	K-12	Required	A, HR, WS	External standard	✓	Students, staff, parents
**Arizona**	✓	-	HS+MS	Encouraged	D, A, HR, WS	-	-	Students
**Arkansas**	-	✓	HS+MS	Required	D, A, HR, WS	Evidence-based	-	Students
**California**	-	-	K-12	Required	D, A, HR, WS, BE	Evidence-based	✓	Students, staff
**Colorado**	✓	✓	HS+MS	Encouraged	A, HR, WS, BE	Evidence-based	✓	Students, staff
**Connecticut**	✓	-	K-12	Required	A, HR, BE	Evidence-based	✓	Students, staff
**Delaware**	✓	-	HS+MS	Required	HR	External standard	-	Students, staff
**D.C.**	✓	✓	K-12	Required	A, HR, WS	Evidence-based	-	Students, staff, parents
**Florida**	-	-	HS+MS	Required	D, HR, WS	External standard	✓	Students, staff
**Georgia**	-	-	HS	Required	Unknown	External standard	✓	Students
**Hawaii**								
**Idaho**	-	-	K-12	Required	A, HR	Evidence-based	-	Students
**Illinois**	✓	-	HS+MS	Required	Unknown	-	-	Students, staff
**Indiana**	-	-	HS+MS	Encouraged	A, WS	External standard	-	-
**Iowa**	-	✓	K-12	Required	A	Evidence-based	-	Students, staff, parents
**Kansas**								
**Kentucky**	-	✓	K-12	Required	D, A, HR, BE	-	✓	Students
**Louisiana**	✓	-	HS+MS	Required	D, A, HR, WS, BE	-	✓	Students, staff, parents
**Maine**	-	-	K-12	Encouraged	HR, BE	-	✓	Students
**Maryland**	-	-	Unknown	Encouraged	A, HR	-	-	Students
**Massachusetts**	-	-	K-12	Required	A, HR, BE	-	✓	Students, parents
**Michigan**	-	-	Unknown	Required	A, HR	Evidence-based	-	Students
**Minnesota**								
**Mississippi**	-	-	K-12	Encouraged	Unknown	-	-	Students
**Missouri**	✓	-	Unknown	Encouraged	A	-	-	-
**Montana**								
**Nebraska**	✓	-	Unknown	Required	D, A, HR, WS	-	-	Students, staff
**Nevada**								
**New Hampshire**	-	-	Unknown	Required	HR	-	-	Staff
**New Jersey**	✓	-	HS+MS	Required	D, HR, WS	-	-	Students
**New Mexico**	-	✓	K-12	Required	D, HR	Evidence-based	-	Students

© 2022 Adhia A et al. *JAMA Pediatrics.*

**New York**								
**North Carolina**	-	-	K-12	Required	HR	Evidence-based	✓	Students, staff
**North Dakota**								
**Ohio**	-	✓	HS+MS	Required	D, A, HR, WS	-	✓	Students, staff
**Oklahoma**	-	-	K-12	Required	HR	External standard	✓	Students, parents
**Oregon**	✓	✓	HS+MS	Required	D, HR, BE	Evidence-based	✓	Students, staff
**Pennsylvania**	✓	-	HS	Encouraged	D, A, HR, WS, BE	External standard	-	Students, staff, parents
**Rhode Island**	✓	-	K-12	Required	D, A, HR, WS	Evidence-based	✓	Students, staff
**South Carolina**								
**South Dakota**								
**Tennessee**	-	-	HS+MS	Required	D, A, HR	Evidence-based	✓	Students, staff
**Texas**	✓	-	K-12	Required	A, HR, BE	External standard	✓	Students, parents
**Utah**								
**Vermont**	-	-	K-12	Required	HR	External standard	-	Students
**Virginia**	-	✓	HS+MS	Required	D	Evidence-based	-	Students
**Washington**								
**West Virginia**	-	-	HS	Required	HR	-	-	Students
**Wisconsin**	-		K-12	Encouraged	HR	-	-	Students
**Wyoming**								

Abbreviation: TDV, teen dating violence.

[a]Grade levels: K-12 = Kindergarten through 12th grade; HS+MS = high school and middle school; HS = high school only; Unknown = not specified

[b]Prevention education content: D = definition of TDV; A = awareness education; HR = healthy relationships; WS = warning signs; BE = bystander education; Unknown = not specified

© 2022 Adhia A et al. *JAMA Pediatrics.*

eTable 2. State Laws Addressing Teen Dating Violence Policy and Response and the Implementation of Teen Dating Violence Policy or Prevention in Secondary Schools in 50 US States and the District of Columbia as of September 30, 2020

	TDV Policy				Response to TDV			Implementation		
**State**	**Develop TDV policy**	**Policy applies[a]**	**External review**	**Who must be trained on policy**	**Investigation requirements**	**Disciplinary consequences**	**Mental health**	**Designated individual**	**Funding provision**	**Non-compliance**
**Alabama**										
**Alaska**	-				-	-	-	✓	✓	✓
**Arizona**	Encouraged	SP	-	-	-	-	-	-	-	-
**Arkansas**	-				-	-	-	-	-	-
**California**	-				-	-	-	-	-	-
**Colorado**	-				-	-	✓	✓	✓	✓
**Connecticut**	Required	SP	✓	Students, staff	-	Develop	-	✓	✓	-
**Delaware**	Required	SP	✓	Students, staff, parents	-	Develop	-	-	-	✓
**D.C.**	Required	SP, ST, SE, E	-	Students, staff, parents	✓	Develop	-	-	-	-
**Florida**	Required	SP, ST, SE	-	Staff	-	Develop	-	-	-	✓
**Georgia**	-				-	-	-	-	-	-
**Hawaii**										
**Idaho**	Required	SP	✓	-	-	-	-	-	-	-
**Illinois**	Required	SP, ST, SE	-	Students, parents	-	-	-	✓	-	-
**Indiana**	Encouraged	SP	-	-	-	-	-	-	-	-
**Iowa**	-				-	-	-	✓	-	✓
**Kansas**										
**Kentucky**	-				-	-	-	-	-	-
**Louisiana**	Required	SP	✓	-	-	Develop	-	-	-	-
**Maine**	-				-	-	-	-	-	-
**Maryland**	-				-	-	-	-	-	-
**Massachusetts**	Required	SP	-	-	-	Develop	-	-	✓	-
**Michigan**	-				-	-	-	-	-	✓
**Minnesota**										
**Mississippi**	-				-	-	-	-	-	-
**Missouri**	-				-	-	-	-	-	-
**Montana**										
**Nebraska**	Required	SP	✓	Staff, parents	-	-	-	-	-	✓
**Nevada**										
**New Hampshire**	-				-	-	-	-	-	-
**New Jersey**	Required	SP, ST, SE	-	Students, staff	Develop	Develop	-	-	-	-
**New Mexico**	-				-	-	-	✓	-	✓
**New York**										

© 2022 Adhia A et al. *JAMA Pediatrics.*

**North Carolina**	-				-	-	-	✓	-	-
**North Dakota**										
**Ohio**	Required	SP, ST, SE, E	✓	Students, staff, parents	✓	Develop	-	-	✓	-
**Oklahoma**	-				-	-	-	-	✓	✓
**Oregon**	Required	SP, ST, SE, E	-	Students, staff, parents	Develop	Develop	-	✓	✓	✓
**Pennsylvania**	Encouraged	SP	-	Staff, parents	-	-	-	-	✓	-
**Rhode Island**	Required	SP, ST, SE	✓	Students, staff, parents	-	Develop	-	✓	-	✓
**South Carolina**										
**South Dakota**										
**Tennessee**	-				-	-	✓	✓	✓	-
**Texas**	Required	SP	✓	Staff	-	-	✓	✓	✓	✓
**Utah**										
**Vermont**	-				-	-	-	✓	-	-
**Virginia**	-				-	-	-	-	-	-
**Washington**										
**West Virginia**	-				-	-	-	-	-	-
**Wisconsin**	-				-	-	-	-	-	-
**Wyoming**										

Abbreviation: TDV, teen dating violence.

[a]Policy applies: SP = school property; ST = school transportation; SE = school event; E = electronic communication

© 2022 Adhia A et al. *JAMA Pediatrics.*
